# Biosourced All-Acrylic ABA Block Copolymers with Lactic Acid-Based Soft Phase

**DOI:** 10.3390/molecules25235740

**Published:** 2020-12-05

**Authors:** Nabil Bensabeh, Ana Jiménez-Alesanco, Ilme Liblikas, Juan C. Ronda, Virginia Cádiz, Marina Galià, Lauri Vares, Olga Abián, Gerard Lligadas

**Affiliations:** 1 Laboratory of Sustainable Polymers, Department of Analytical Chemistry and Organic Chemistry, University Rovira i Virgili, 43007 Tarragona, Spain; nabil.bensabeh@urv.cat (N.B.); juancarlos.ronda@urv.cat (J.C.R.); virginia.cadiz@urv.cat (V.C.); marina.galia@urv.cat (M.G.); 2 Institute for Biocomputation and Physics of Complex Systems (BIFI), Joint Units IQFR-CSIC-BIFI, and GBsC-CSIC-BIFI, Universidad de Zaragoza, 50018 Zaragoza, Spain; ajimenez@bifi.es (A.J.-A.); oabifra@unizar.es (O.A.); 3 Institute of Technology, University of Tartu, Nooruse 1, 50411 Tartu, Estonia; ilme.liblikas@ut.ee (I.L.); lauri.vares@ut.ee (L.V.); 4 Instituto Aragonés de Ciencias de la Salud (IACS), 50009 Zaragoza, Spain; 5 Instituto de Investigación Sanitaria de Aragón (IIS Aragon), 50009 Zaragoza, Spain; 6 Centro de Investigación Biomédica en Red en el Área Temática de Enfermedades Hepáticas Digestivas (CIBERehd), 28029 Madrid, Spain; 7 Departamento de Bioquímica y Biología Molecular y Celular, Universidad de Zaragoza, 50005 Zaragoza, Spain

**Keywords:** block copolymers, renewable resources, RAFT, alkyl lactate, PSA

## Abstract

Lactic acid is one of the key biobased chemical building blocks, given its readily availability from sugars through fermentation and facile conversion into a range of important chemical intermediates and polymers. Herein, well-defined rubbery polymers derived from butyl lactate solvent were successfully prepared by reversible addition–fragmentation chain transfer (RAFT) polymerization of the corresponding monomeric acrylic derivative. Good control over molecular weight and molecular weight distribution was achieved in bulk using either monofunctional or bifunctional trithiocarbonate-type chain transfer agents. Subsequently, poly(butyl lactate acrylate), with a relative low *T*_g_ (−20 °C), good thermal stability (5% wt. loss at 340 °C) and low toxicity was evaluated as a sustainable middle block in all-acrylic ABA copolymers using isosorbide and vanillin-derived glassy polyacrylates as representative end blocks. Thermal, morphological and mechanical properties of copolymers containing hard segment contents of <20 wt% were evaluated to demonstrate the suitability of rubbery poly(alkyl lactate) building blocks for developing functional sustainable materials. Noteworthy, 180° peel adhesion measurements showed that the synthesized biosourced all-acrylic ABA copolymers possess competitive performance when compared with commercial pressure-sensitive tapes.

## 1. Introduction

Nature uses molecular self-assembly to create precision nanostructures and build large constructs through hierarchical assembly [1,2]. Inspired by these motifs, considerable efforts have been undertaken to recreate such concepts using synthetic block copolymers fashioned from two or more chemically dissimilar components that are covalently-bonded into a single molecule [3]. Linear ABA block copolymers with a soft middle block and hard minority end blocks are of high utility and interest for a wide variety of applications, ranging from adhesives to clothing, automotive and biomedical components due to their remarkable (re)processable structures [4]. Among them, all-acrylic systems offer advantages to the current gold-standard systems based on poly(styrene-*block*-isoprene-*block*-styrene) and poly(styrene-*block*-butadiene-*block*-styrene) copolymers, in terms of wider service temperature range, improved optical transparency and stability to UV light [5,6]. In addition, acrylate-based triblocks may exhibit excellent performance as pressure-sensitive adhesives (PSAs), even without the incorporation of additives such as tackifiers and plastisizers [7]. 

Making the most of reversible deactivation radical polymerizations techniques [8], the rich assortment of (meth)acrylate monomers from biosourced feedstocks offers an attractive palette of rubbery and glassy polymers for the design of innovative sustainable all-acrylic ABA-type thermoplastic elastomers (TPEs) with competitive properties. For instance, poly(lauryl methacrylate) (*T*_g_ ≈ −46 °C) as the rubbery segment and poly(acetylsalicylic ethyl metacrylate) (*T*_g_ ≈ 53 °C) as the glassy segment were combined to build up ABA copolymer architectures exhibiting elastomeric behavior at room temperature [9]. Biosourced acrylic monomers derived from glucose [10], isosorbide [11], itaconic acid imides [12] and rosin [13], as well as aromatic lignin derivatives [14], have been shown to provide useful glassy components for developing sustainable elastomeric and adhesive materials based on ABA block copolymers. In most of these studies, poly(butyl acrylate) (*T*_g_ ≈ −50 °C) was chosen as the rubbery midblock, since it is a conventional component of acrylic ABA copolymers with tunable properties according to block lengths and molecular composition. Although it is feasible to derive nBA from bio-sourced acrylic acid and butan-1-ol [15], the search for alternative rubbery blocks from biobased feedstocks represents a key step forward towards improving the material’s sustainability. In this regard, fatty acids acrylic derivatives [16], dialkyl itaconates [12] and tetrahydrogeraniol derivatives [17] are only selected examples on how to create renewable triblock copolymers with biobased soft phase without compromising performance.

Lactic acid is one of the top value added biomass derived platform chemicals, given its ready availability from sugars through fermentation and its facile conversion into a number of important derivatives [18,19,20]. Among them, polylactide (PLA), most commonly synthesized by the ring-opening polymerization of the cyclic dimer of lactic acid, is a good alternative replacement for the traditional petroleum-sourced polystyrene glassy end-blocks in ABA TPEs, due to their high modulus characteristics [21]. The potential of alkyl lactate esters, e.g., methyl, ethyl and butyl, goes beyond their well-recognized use as eco-friendly solvents [22], as their secondary alcohol offers a simple access to monovinyl derivatives prone to polymerization by radical mechanisms [23,24,25,26]. In this regard, our group reported that poly(alkyl lactate ester) (meth)acrylates with controlled molecular weight, narrow molecular weight distribution and high end-group fidelity are also easily accessible by Cu(0) wire-mediated single-electron transfer living radical polymerization (SET-LRP) [27,28]. In a more recent effort, the different water solubility of poly(ethyl lactate acrylate)s (hydrophobic and water-insoluble) and poly(*N*,*N*-dimethyl lactamide acrylate) (hydrophilic and water-soluble) was exploited to design amphiphilic AB block copolymers from neoteric lactic acid-based solvents, i.e., ethyl lactate and *N*,*N*-dimethyl lactamide, that could self-assemble in aqueous solution to form nanoparticles with different morphologies including large-compound micelles and vesicles [29].

Herein, we report on the preparation of all-acrylic ABA copolymers with a lactic acid acrylic derivative as an elastomeric building block. A biobased acrylic monomer, l-butyl lactate acrylate (BuLA), has been synthesized from the corresponding alkyl lactate, which is used as a solvent and a dairy-related flavoring agent approved as a food additive by the US Food and Drug Administration. This monomer was conceived to impart a lactic acid-based sustainable soft segment into ABA-type block copolymers. RAFT polymerization of the BuLA monomer allowed the preparation of fine-tuned all-acrylic triblock copolymers combining soft poly(BuLA) segments with isosorbide and vanillin-derived glassy end blocks. The ABA architecture were investigated for the fidelity of phase-separation in the bulk, and were initially examined for their mechanical and adhesion properties. The results reported here demonstrate that biosourced and non-toxic acrylic derivatives of alkyl lactate esters can complement the classic alkyl acrylate palette for developing sustainable materials with assembly properties that can be utilized in a wide variety of applications.

## 2. Results and Discussion

### 2.1. BuLA Monomer Synthesis and RAFT Polymerization Model Studies

**Figure 1:**
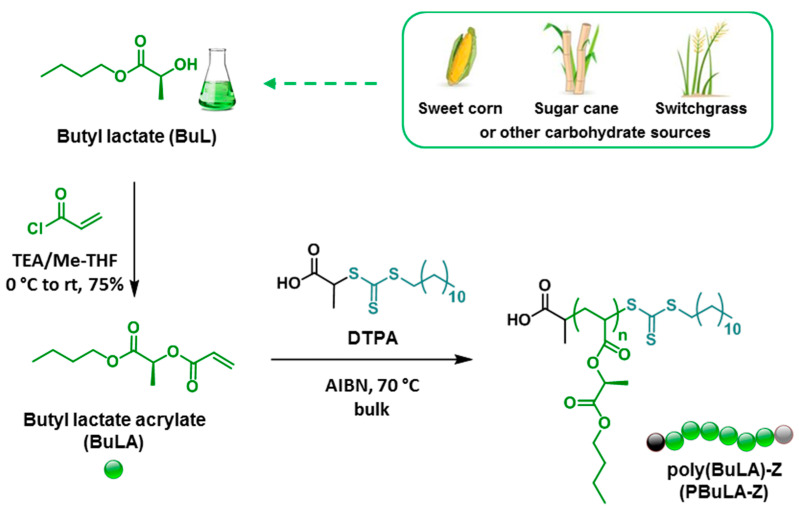
Synthesis of BuLA from biorenewable BuL solvent and their bulk reversible addition–fragmentation chain transfer (RAFT) polymerization using a monofunctional chain transfer agent (CTA), namely 2-(dodecylthiocarbonothioylthio)propionic acid (DTPA) to yield PBuLA-Z.

**Figure 2:**
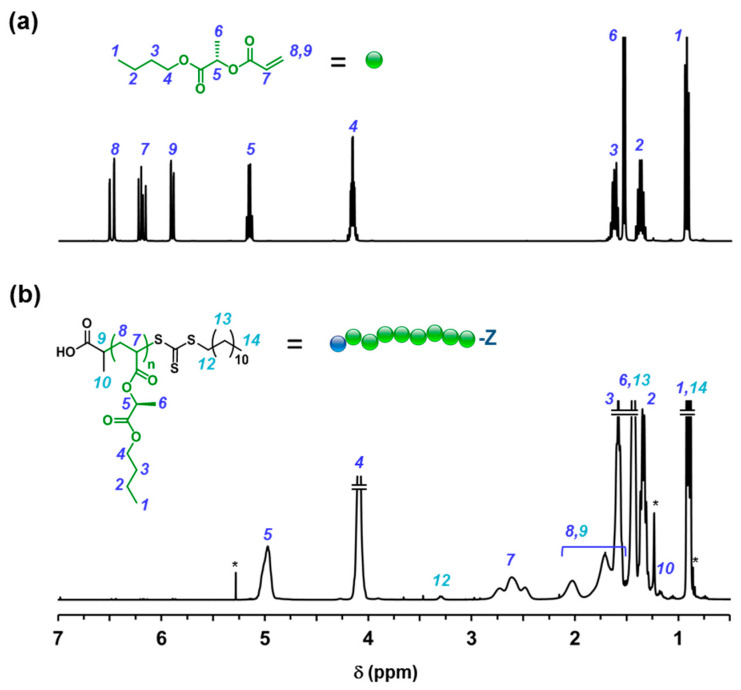
^1^H NMR spectra of (**a**) BuLA and (**b**) PBuLA-Z (*M*_n,GPC_ = 10,900, *M*_w_/*M*_n_ = 1.24) in CDCl_3_. ^1^H NMR resonances from “grease” impurities are indicated with *.

As reported in previous publications, alkyl lactate solvents are appealing chemicals to prepare biorenewable acrylic/methacrylic polymers [23,24,25,26,27,28,29]. Here, BuLA was prepared from l-butyl lactate (BuL) by acylation with acryloyl chloride in the presence of base using a solvent with low environmental impact such as Me-THF (Figure 1). After vacuum distillation in the presence of hydroquinone to minimize polymer formation, BuLA was isolated as a colorless liquid in high yield (75%). The structure of BuLA was verified by NMR spectroscopy. Figure 2a shows ^1^H NMR spectrum of BuLA, in which the three acrylic protons appear between 6.5 and 5.8 ppm. The characteristic BuL methine proton [CH(CH_3_)O], at around 4.3 ppm, shifted to 5.6 ppm after the formation of the ester group. The ^13^C NMR spectrum was also consistent with that expected for BuLA (see experimental section).

**Figure 3:**
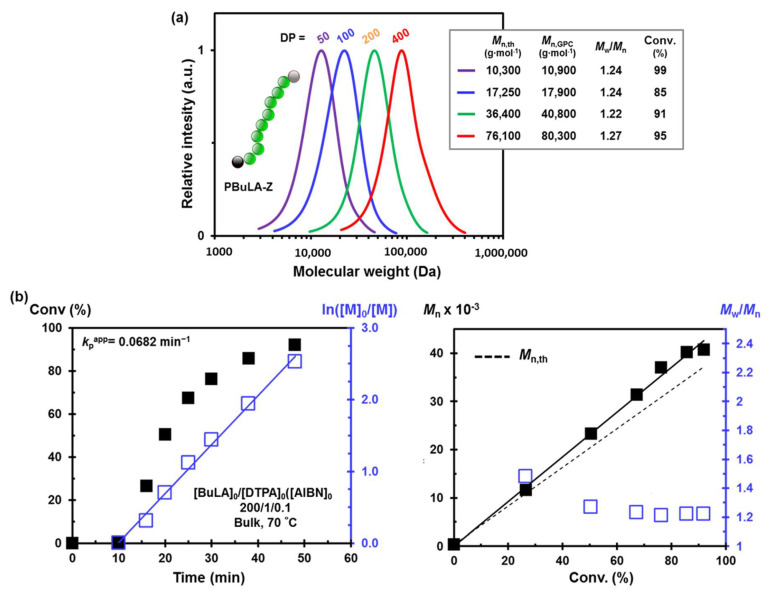
Bulk RAFT polymerization of BuLA with DTPA at 70 °C. (**a**) GPC traces of PBuLA-Z prepared at different degrees of polymerization (DPs), ranging from 50 to 400. The inset contains *M*_n,th,_ *M*_n,GPC,_ *M*_w_/*M*_n_, and monomer conversion values; and (**b**) evolution of monomer conversion and semilogarithmic kinetic plot of ln([M]_0_/[M]) versus time (left panel) and evolution of experimental *M*_n,GPC_ and *M*_w_/*M*_n_, based on the calibration by PMMA standards, with the monomer conversion (right panel). Reaction conditions for (b): BuLA = 1 mL, [BuLA]_0_/[DTPA]_0_/[AIBN]_0_ = 200/1/0.1 at 70 °C.

After monomer synthesis, we investigated its controlled polymerization by using the RAFT technique, on account of its ability to form polymers with predicted molecular weight and relatively low molecular weight distribution (MWD) in a green fashion [30]. Figure 1 depicts the RAFT polymerization of BuLA at 70 °C in bulk, using 2,2′-azobisisobutyronitrile (AIBN) and 2-(dodecylthiocarbonothioylthio)propionic acid (DTPA) as radical initiator and chain transfer agent (CTA), respectively. A series of polymerizations were conducted at different [BuLA]_0_/[DTPA]_0_ ratios (50 to 400) to target various polymer chain lengths. PBuLAs grown in one direction, defined as PBuLA-Z, with polydispersity (*M*_w_/*M*_n_) ranging from 1.22–1.27 and gel permeation chromatography (GPC)-determined molecular weight (*M*_n,GPC_) as high as *M*_n,GPC_ = 80,300 g/mol could be obtained after 2 h (Figure 3a). In all cases, the bulk RAFT process proceeded to high monomer conversions (>90%) and yielding polymers with *M*_n,GPC_ close to the theoretical values (*M*_n,th_), calculated from monomer conversion assuming complete consumption of CTA. The small discrepancy in the *M*_n,GPC_ and *M*_n,th_ data may be attributed to differences in hydrodynamic volume between PBuLA-Z and PMMA standards used for calibration.

**Figure 4:**
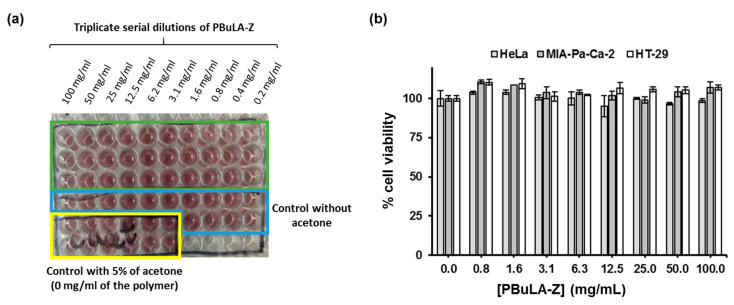
(**a**) The plates for cytotoxic assays of PBuLA-Z (*M*_n,GPC_ = 11,000 Da, *M*_w_/*M*_n_ = 1.23) tested in HeLa cells; and (**b**) CellTiter cell viability assay results for the same polymer at different concentrations assessed in HeLa, MIA-Pa-Ca-2 and HT-29 cell lines.

As a representative example, purified low molar mass PBuLA-Z prepared at degree of polymerization (DP) of 50 (*M*_n,GPC_ = 10,900 Da, *M*_w_/*M*_n_ = 1.24) was characterized by ^1^H NMR (Figure 2b). After polymerization, the olefinic protons, i.e., 5.8–6.5 ppm, in the BuLA monomer were incorporated into the backbone of the new vinylic polymer (i.e., 1.9–3.0 ppm). Moreover, the peak at ≈ 3.3 ppm, attributed to the CH_2_-S protons of RAFT CTA groups located at the ω-chain end of the polymer, supports the “livingness” of the synthesized polymer. Next step, we further investigated the kinetics of the polymerization at a DP of 200. As can be seen in Figure 3b (left panel), after a 10 min induction period attributed to the difficulty in deoxygenating a highly viscous reaction mixture, the polymerization achieved ≈ 90% conversion in less than 1 h. The linear evolution of ln[M]_0_/[M] versus reaction time suggested a constant concentration of active species throughout the entire RAFT process. Moreover, as expected for a controlled polymerization, molecular weights increased linearly with conversion retaining narrow MWD (*M*_w_/*M*_n_ ≈ 1.25) even at high values (right panel). Prior to conducting block copolymerization studies, we evaluated the cytotoxic effect of PBuLA-Z RAFT homopolymer by treating HeLa, HT-29 and MIA-Pa-Ca-2 cell lines with different doses of the homopolymer dissolved in the culture medium (Figure 4). Figure 4a depicts the CellTiter plates for different concentrations of PBuLA-Z (*M*_n,GPC_ = 11,000 Da, *M*_w_/*M*_n_ = 1.23) for HeLa cells culture. CellTiter colorimetric assay was used for determining cell viability and cellular proliferation after the addition of test RAFT polymer. This assay determines the number of viable cells due to the bioreduction of the MTS tetrazolium compound (Owen’s reagent) into a colored formazan product accomplished by NADPH or NADH produced by dehydrogenase enzymes in metabolically active cells. Therefore, color formation can be a useful marker of viable cells. Notably, the cytotoxicity analysis using the CellTiter assay showed that 24 h exposure of the cells to up to 100 mg·mL^−^^1^ at 37 °C did not reduce cell viability. Hence, these results further support the use of PBuLA polymers as a soft and hydrophobic building block in well-defined copolymer synthesis employed in a wide range of applications, including biomedical devices and adhesives [31].

### 2.2. Bifunctional PBuLA-2Z macro-CTA via bulk RAFT Polymerization

**Figure 5:**
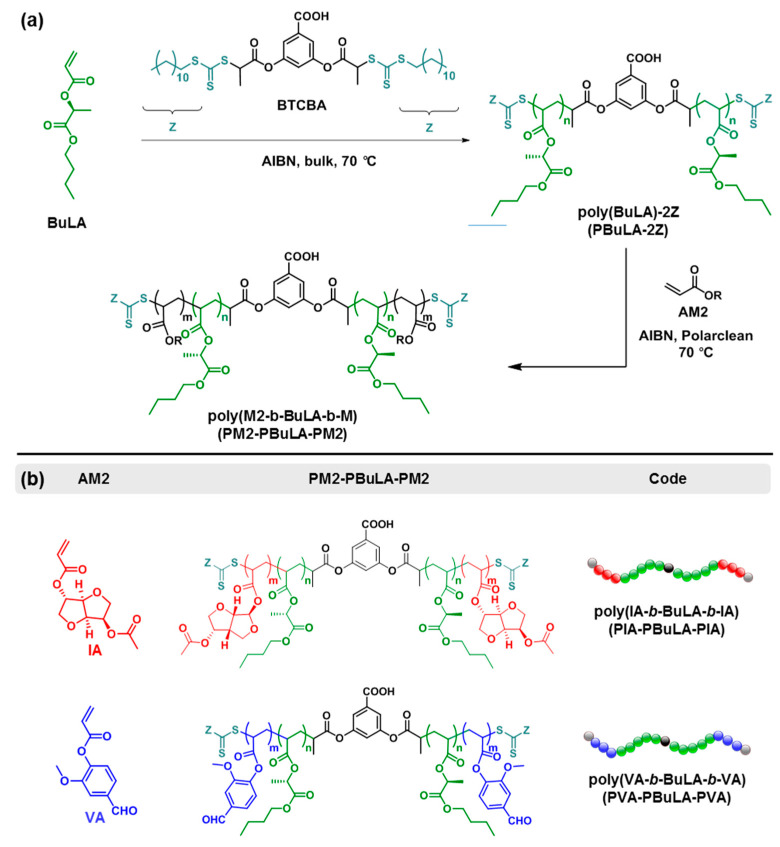
(**a**) Two-step RAFT synthesis of ABA block copolymers with lactic acid-based middle block using AM2 monomer to build up end blocks and BTCBA as a CTA. (**b**) Chemical structures of AM2 acrylic monomers (IA and VA) and ABA block copolymers (PIA-PBuLA-PIA and PVA-PBuLA-PVA) studied herein.

With the final goal to prepare ABA block copolymers with PBuLA as the soft middle block, we used a commercially available bifunctional CTA, namely 3,5-bis(2-dodecylthiocarbonothioylthio-1-oxopropoxy)benzoic acid (BTCBA), to prepare bifunctional PBuLA macroCTAs (defined as PBuLA-2Z) through divergent RAFT polymerization of BuLA at 70 °C in bulk (see first reaction step in Figure 5a). 

**Figure 6:**
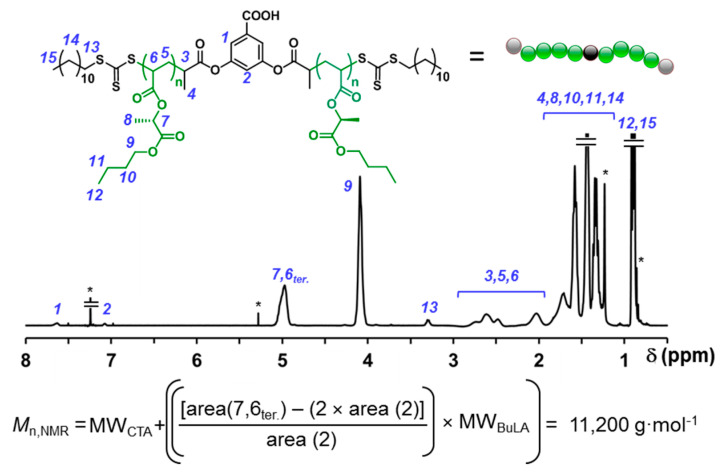
^1^H NMR spectrum PBuLA-2Z (*M*_n,GPC_ = 10,900, *M*_w_/*M*_n_ = 1.24) in CDCl_3_. ^1^H NMR resonances from “grease” impurities and residual solvents are indicated with *.

Initially, we targeted a low molar mass polymer (DP = 50, *M*_n,th_ = 10,830 g·mol^−1^) to collect structural information about the “livingness” of the system when performing divergent chain growth. Under these conditions, the RAFT polymerization of BuLA achieved 99% monomer conversion in 2 h. BTCPA controlled the polymerization of BuLA and yielded PBuLA-2Z macroinitiator with *M*_n,GPC_ of 10,500 and *M*_w_/*M*_n_ of 1.24 (Supplementary Materials Figure S1). ^1^H NMR analysis of the isolated polymer clearly revealed the presence of CTA residues at both the polymer core and chain ends, e.g., signals 7.68 and 7.13 ppm for aryl mid group protons and 3.33 ppm for the α methylene protons of the trithiocarbonate end groups (Figure 6). Hence, the ratio of integration of these signals to the methylene (CH_2_-O) peak of the butyl lactate repeating units enabled to determine *M*_n,NMR_ (11,200 g·mol^−1^) which was in good agreement with *M*_n,th_ and *M*_n,GPC_ (11,700 g·mol^−1^ and 11,000 g·mol^−1^, respectively). Note that the *M*_n,NMR_ was only about 5% above *M*_n,th_. Thus, within the experimental error of ^1^H NMR measurement, these results support good control of the bulk RAFT polymerization. Next, we pushed this polymerization system to higher DPs (100–400) under strictly identical reaction conditions, as higher molar mass polymers are required to prepare ABAs with competitive performance. Also in this case, high conversions (>90%) were achieved. 

**Figure 7:**
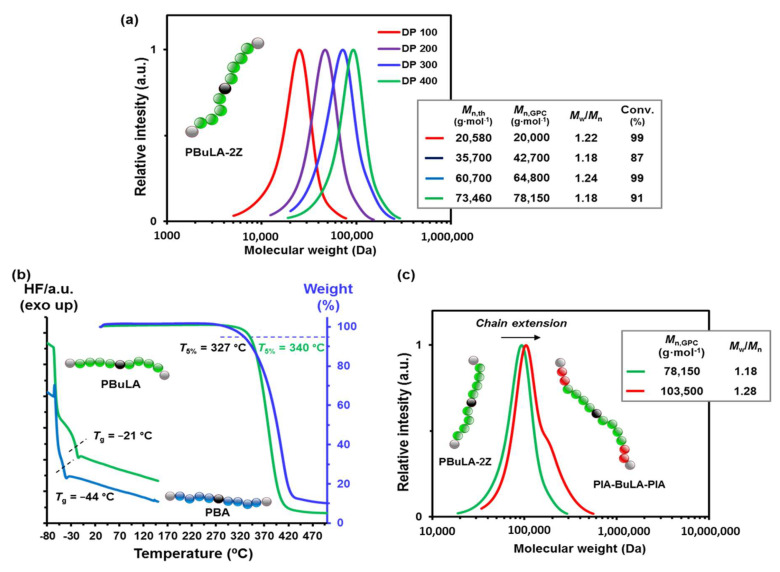
(**a**) Gel permeation chromatography (GPC) traces of PBuLA-2Z prepared at different DPs ranging from 50 to 400 by the bulk RAFT polymerization of BuLA with BTCBA at 70 °C (The inset contains *M*_n,th,_ *M*_n,GPC,_ *M*_w_/*M*_n_, and monomer conversion values); (**b**) thermal characterization by differential scanning calorimetry (DSC) and thermogravimetric analysis (TGA) analyses of PBuLA-2Z (*M*_n,GPC_ = 78,150 g·mol^−1^) and PBA (*M*_n,GPC_ = 97,900 g·mol^−1^); and (**c**) GPC traces of the chain extension of PBuLA-2Z macro-RAFT agent with IA monomer at 70 °C in Rhodiasolv^®^ PolarClean solvent. The inset contains *M*_n,GPC_ and *M*_w_/*M*_n_ values.

As shown in Figure 7a, the successful synthesis of PBuLA-2Z macro-CTAs with molecular weight as high as *M*_n,GPC_ = 78,150 g·mol^−1^ was demonstrated by unimodal GPC traces and *M*_n,GPC_ values very close to the theoretical values and narrow *M*_w_/*M*_n_ values (<1.25). Good candidates for the soft middle block in triblock TPEs must have a glass transition temperature (*T*_g_) lower than their application temperature, in order to provide flexibility or tacky characteristics to the final materials. 

Differential scanning calorimetry (DSC) analysis of PBuLA-2Z (*M*_n,GPC_ = 78,150) revealed a *T*_g_ value (indicated by dashed line in the green trace) well below room temperature (*T*_g_ = −21 °C) (Figure 7b). The reduction of molar mass from *M*_n,GPC_ = 78,150 g·mol^−1^ to *M*_n,GPC_ = 20,000 g·mol^−1^ did not significantly influence the value of *T*_g_ (data not shown). Note that the *T*_g_ value for PBuLA-2Z is 23 °C above that of poly(butyl acrylate) (PBA) of approximately the same *M*_n_ prepared by RAFT (Figure 7b). This result can be attributed to the presence of two polar carbonyl units per repeating unit, which decreases segmental motion. Thermogravimetric analysis (TGA) also revealed that PBuLA-2Z is also highly competitive in terms of thermal stability (Figure 7b). The thermal stability of PBuLA-2Z was slightly higher, with a 5% degradation temperature of 340 °C (327 °C for PBA). Overall, these results support the use of PBuLA as an innovative candidate soft block in triblock copolymer-based ABA-type TPEs. 

### 2.3. ABA Block Copolymers via RAFT in Rhodiasolv^®^ PolarClean Solvent

**Figure 8:**
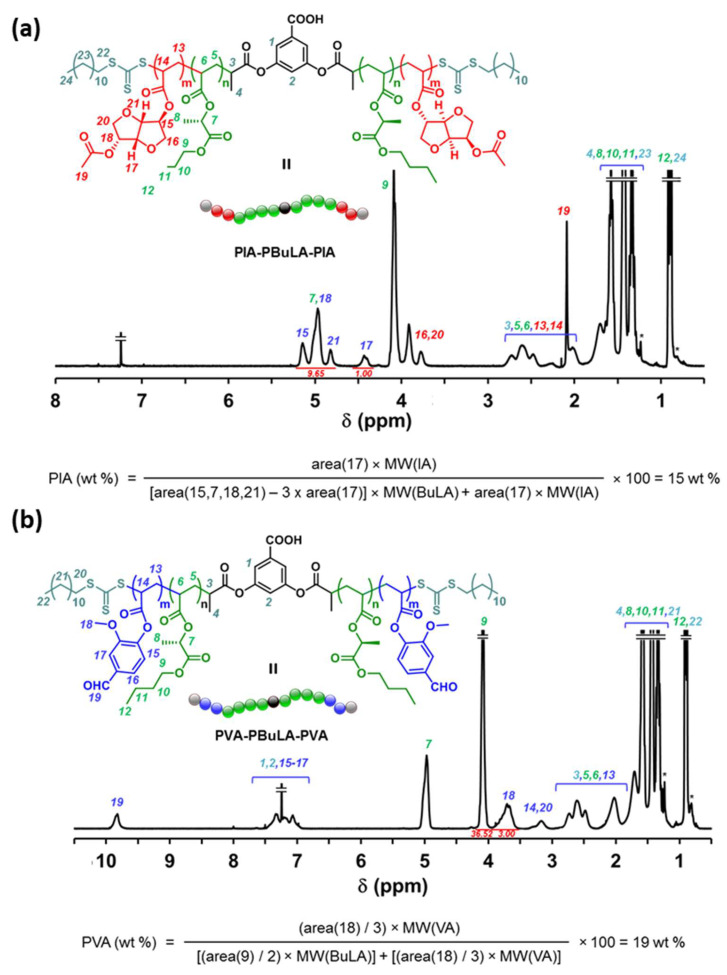
^1^H NMR spectra of: (**a**) PIA-PBuLA-PIA (*M*_n,GPC_ = 103,500 g·mol^−1^, *M*_w_/*M*_n_ = 1.28); and (**b**) PVA-PBuLA-PVA (*M*_n,GPC_ = 110,150 g·mol^−1^, *M*_w_/*M*_n_ = 1.38) triblocks in CDCl_3_. ^1^H NMR resonances from “grease” impurities are indicated with *.

Using PBuLA-2Z (*M*_n,GPC_ = 78,150 g·mol^−1^) as macroCTA, we subsequently studied the incorporation of glassy external segments at the chain ends of PBuLA in a divergent fashion via RAFT polymerization with a second acrylic monomer AM2 (see the second reaction step in Figure 5a,b). Two sustainable triblock copolymers were targeted using poly(vanillin acrylate) (PVA) and a poly(isosorbide acrylate) (PIA) as hard blocks. The chemical structure of both biosourced acrylic monomers (IA, i.e., isosorbide 2-acrylate-5-acetate, and VA, i.e., vanillin acrylate) and the targeted ABA block copolymers (PVA-PBuLA-PVA and PIA-PBuLA-PIA) is shown in Figure 5b. IA and VA were prepared from isosorbide and vanillin, respectively, using previously reported synthetic procedures [32,33,34]. Unfortunately, the chain extension step required the use of solvent to build the pursued triblocks, as reaction mixtures were not homogeneous at the polymerization temperature (70 °C). Although recent advances in waterborne processes are highly attractive [35], the search for eco-friendly solvents as an alternative to conventional petroleum-derived solvents is also desirable. For this purpose, after control experiments that will be reported in a forthcoming publication, block copolymer syntheses were performed in ethyl-5-(dimethylamido)-2-methyl-5-oxopentanoate (Rhodiasolv^®^ PolarClean) [36]. To our knowledge, this eco-friendly water-soluble and polar solvent was never used as green reaction media in controlled radical polymerization. Hence, chain extension of PBuLA-2Z (*M*_n,GPC_ = 75,340 g·mol^−1^) with IA at 70 °C under the reaction conditions of [IA]_0_/[PBuLA-2Z]_0_/[AIBN]_0_ = 109/1/0.2 in PolarClean was afforded the PIA-PBuLA-PIA triblock. The conversion of IA monomer was calculated to be 86%, using ^1^H NMR analysis prior to purification of the polymer (Figure S2). A clear shift to higher molar masses in the SEC trace indicated successful chain extension (Figure 7c). Note that a high molar mass shoulder was present in the GPC trace of the ABA copolymer, likely due to termination by combination at high conversion. Nevertheless, the molar mass distribution was relatively narrow (*M*_w_/*M*_n_ = 1.28) suggesting that the chain extension occurs, retaining substantial control. As depicted in Figure S3, chain extension PBuLA-2Z with VA also resulted in success to furnish PVA-PBuLA-PVA triblock (78% conversion, *M*_n,GPC_ = 110,150 g·mol^−1^). Both triblocks were subsequently characterized by ^1^H NMR to determine their compositions (Figure 8). For instance, as indicated in Figure 8a, the composition of block copolymer PIA-PBuLA-PIA could be determined from the integration of signals at 4.3–4.5 ppm from H_17_ corresponding to PIA units and the integration of the signals at 4.7–5.3 ppm from PBuLA units, after subtracting the peak area contributed by the PIA units (H_15_, H_18_, and H_21_) in this region. Thus, the obtained PIA-PBuLA-PIA triblock had a PIA (hard block) weight percentage of 15 wt%, as determined via ^1^H NMR spectroscopy, which is comparable to the theoretical 18 wt% value determined from monomer conversion. As depicted in Figure 8b, the hard block content for the PVA-PBuLA-PVA copolymer was slightly higher (19 wt% PVA), but also comparable with the theoretical value calculated from conversion (21 wt%). Note that the hard block content in these representative copolymers is consistent with typical hard block composition for triblock copolymers used for pressure-sensitive adhesive (PSA) applications, where high tacky materials are pursued [10,14]. 

### 2.4. ABA Block Copolymers Characterization: Thermal, Mechanical, and Ahesive Properties

**Figure 9:**
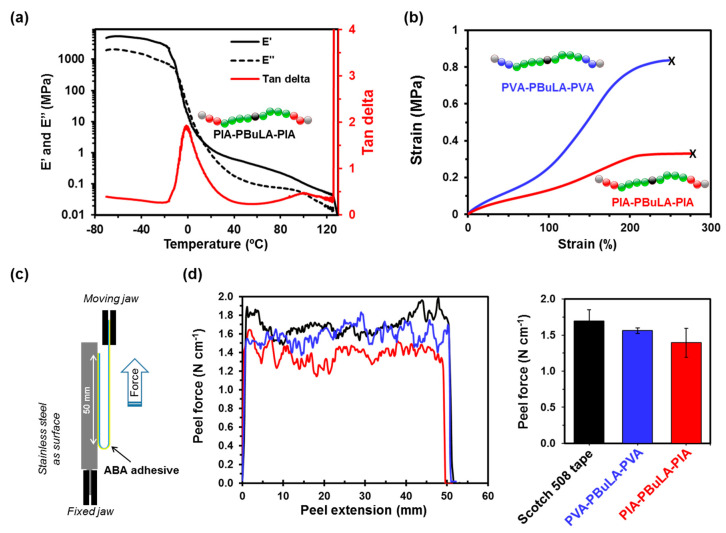
(**a**) Dynamic tensile storage (E’) and loss (E’’) moduli and tan (δ = E’’/E’) as a function of temperature of PIA-PBuLA-PIA triblock; (**b**) stress-strain curves of PIA-PBuLA-PIA and PVA-PBuLA-PVA; (**c**) schematic design of 180° peel test; and (**d**) 180° peel force of PIA-PBuLA-PIA, PVA-PBuLA-PVA and Scotch 508 tape.

The microstructure and viscoelastic properties of the synthesized triblock copolymers were studied by DSC and dynamic mechanical thermal analysis (DMTA). DSC analysis of both synthesized triblocks revealed a low *T*_g_ for the PBuLA midblock at about −20 °C, although no discernible signals corresponding to the *T*_g_ of the glassy end blocks were observed, likely due to the lack of a DSC signal based on the low weight fraction of hard block (Figure S4) [9,10,11,14]. However, the presence of midblock *T*_g_ near that of the homopolymer suggests that PBuLA segment is not mixed with either PIA or PVA segments. Note that the *T*_g_ of PIA and PVA homopolymer glassy blocks was expected to appear around 80 °C, as indicated DSC analysis of model homopolymers also prepared by RAFT (dashed lines in Figure S4). Next, both copolymers were submitted to DMTA (Figure 9a and Figure S5). DMTA revealed a relaxation process at around 0 °C, corresponding to the glass-rubber transition of the PBuLA soft phase in PIA-PBuLA-PIA and PVA-PBuLA-PVa triblocks, as indicated by a stepwise decrease in the storage (E’) and loss (E’’) moduli, as well as a peak in tan δ. The presence of a second tan δ peaks at around 100 °C in the case of PIA-PBuLA-PIA triblock, and at 130 °C for the copolymers integrating PVA, which confirmed the soft-hard-soft microstructure configuration of the synthesized triblock copolymers [37]. Note also that E’ (25 °C) for PVA-PBuLA-PVA triblock was slightly higher than that observed for PIA-PBuLA-PIA (1.3 MPa versus 1.0 MPa). This can be attributed to the higher weight percentage of hard block for PVA-PBuLA-PVA (19 wt% of PVA) in comparison to PIA-PBuLA-PIA (15 wt% of PVA). 

Next step, mechanical properties of both triblocks were measured by monotonic tensile tests on polymer films prepared by a hot-press technique (Figure 9b). Both systems exhibited comparable elongation at a break at around 250%. However, PVA-PBuLA-PVA with 19 wt% hard block content demonstrated improved tensile strength (0.83 versus 0.33 MPa). Nevertheless, it is noteworthy that the relatively low tensile strength (<1 MPa) of these materials points toward good adhesion performance (*vide infra*) [10]. TGA was used to evaluate the thermal stability of the synthesized copolymers (Figure S6), and no significant differences were found between them. TGA under an inert atmosphere showed 5 wt% loss temperatures of PVA-PBuLA-PVA and PIA-PBuLA-PIA to be 386 °C and 389 °C, respectively. Hence, these polymers can be processes at elevated temperatures without degradation, and could therefore be used in practical applications demanding high temperatures, e.g., hot melt adhesives [37]. Finally, the adhesion properties of the synthesized all-acrylic ABA block biopolymers with a lactic acid-based elastomeric phase were evaluated. A 30 wt% solution of PVA-PBuLA-PVA and PIA-PBuLA-PIA was spread on a polyethylene terephthalate (PET) film [10,14].

After complete evaporation of the solvent at room temperature, the coated PET films were adhered onto a stainless steel plate, and the peel resistance was measured by pulling the adhered films off the plate at an angle of 180° (Figure 9c). Data for both polymers is presented in Figure 9d, and compared with a commercial Scotch 508 tape. The 180° peel strength for PVA-PBuLA-PVA and PIA-PBuLA-PIA was about 1.5 N·cm^−1^, which is comparable to the tested commercial tape. All the investigated samples had adhesive failure, leaving no residue on the adherend. It is important to mention that both synthesized triblock copolymers were tested without the addition of tackifier or additive. Hence, taking into account that no optimization studies were conducted, these results suggest that the all-acrylic ABA copolymers with a lactic acid-based elastomeric building block are promising materials for PSA applications, and open the door to further investigations in this line.

## 3. Materials and Methods 

### 3.1. Materials

3,5-Bis(2-dodecylthiocarbonothioylthio-1-oxopropoxy)benzoic acid (BTCBA, 98%), 2-(Dodecylthiocarbonothioylthio)propionic acid (DTPA, 98%), acryloyl chloride (≥97%), triethylamine (TEA, ≥99%), 2-methyltetrahydrofuran (Me-THF, 99.5%), n-Butyl acrylate (BA, 99%), butyl l-lactate (98%) were all purchased from Merck KGaA (Darmstadt, Germany) and used as received unless otherwise specified. Thus, BA was passed through a short column of basic Al_2_O_3_ prior to use in order to remove the radical inhibitor and both TEA and Me-THF were distilled prior to use from CaH_2_ and sodium/benzophenone, respectively. 2,2′-Azobis(2-methylpropionitrile) [AIBN, ≥98%, Fluka (Buchs, Switzerland)] was recrystallized three times from methanol before further use. Deuterated chloroform (CDCl_3_) was purchased from Eurisotop, and ethyl-5-(dimethylamido)-2-methyl-5-oxopentanoate (Rhodiasolv^®^ PolarClean) was kindly donated by Solvay (Aubervilliers, France). Isosorbide acetate, i.e., isosorbide 2-acrylate-5-acetate and vanillin acrylate monomers were prepared following previously reported synthetic procedures [32,33,34].

### 3.2. Methods

400 MHz (for ^1^H) and 100.6 MHz (for ^13^C) NMR spectra were recorded on a Varian VNMR-S400 NMR instrument at 25 °C in CDCl_3_ with tetramethylsilane (TMS) as an internal standard. The number-average molecular weight (*M*_n_) and polydispersity (*M*_w_/*M*_n_) of the synthesized polymers were obtained via GPC using an Agilent 1200 series system (Agilent Technologies Inc., Santa Clara, CA, USA) equipped with three serial columns (PLgel 3 μm MIXED-E, PLgel 5 μm MIXED-D and PLgel 20 μm from Polymer Laboratories) and an Agilent 1100 series refractive-index detector. THF HPLC grade (Panreac Química S.L.U., Barcelona, Spain) was used as eluent at a flow rate of 1.0 mL/min. The calibration curves for GPC analysis were obtained with poly(methyl methacrylate) (PMMA) standards purchased from American Polymer Standards (Mentor, OH, USA). The molecular weights were calculated using the universal calibration principle and Mark-Houwink parameters. The glass transition temperature (*T*_g_) of all polymers was determined on the second heating/cooling ramps using DSC measurements conducted on a Mettler DSC3+ thermal analyzers (Mettler-Toledo S.A.E., Barcelona, Spain) using N_2_ as a purge gas (50 mL/min) at scanning rate 20 °C/min in the −80 to 150 °C temperature range. Calibration was based on an indium standard (heat flow calibration) and an indium-lead-zinc standard (temperature calibration). Thermal stability studies were carried out on a Mettler TGA2/LF/1100 (Mettler-Toledo S.A.E., Barcelona, Spain) with N_2_ as a purge gas at flow rate of 50 mL/min. The studies were performed in the 30–600 °C temperature range at a heating rate of 10 °C/min. ESI MS were run on a chromatographic system Agilent G3250AA liquid chromatography coupled to 6210 Time of Flight (TOF) mass spectrometer from Agilent Technologies Inc. (Santa Clara, CA, USA) with an ESI interface. Dynamic mechanical analysis of all polymers was performed on a DMA Q800 (TA Instruments, New Castle, DE, USA) in tension configuration. Rectangular-shaped specimens (5 mm length, 5 mm width, 0.12 mm thickness) were prepared by compression molding into a rectangular steel mould (20 mm 5 mm 1.2 mm) between two parallel steel plates on a Specac Atlas Manual Hydraulic Press (Specac Ltd, Orpington, UK) at 100 °C, with an applied load of 4 tons for 30 min. Then, the rectangular specimen was further heated in the range of −70 to 200 °C using a heating rate of 3 °C/min and a fixed frequency of 1 Hz. For tensile testing, the lower grip was stationary, and the upper grip was raised at a force ramp rate of 1 N/min to obtain tensile strength and elongation at break of polymer at 27 °C. The peel strength analysis was performed on an Instron 5965 (Instron, Bucks, UK) at a peel rate of 300 mm·min^−1^.

### 3.3. Synthesis of BuLA Monomer

Butyl l-lactate (20.0 g, 0.14 mol) and anhydrous triethylamine (21.4 g, 0.21 mol) were dissolved in dry 2-methyltetrahydrofuran (Me-THF; 50 mL) under a constant flow of argon. After the solution was stirred for 30 min at 0–5 °C, acryloyl chloride (14.8 g, 0.16 mol) dissolved in dry Me-THF (50 mL) was added dropwise. The reaction was allowed to proceed for 24 h at room temperature. The reaction mixture was then filtered and Me-THF was removed under reduced pressure. The resulting residue was dissolved in 150 mL of diethyl ether, and the solution was subsequently washed with HCl 1M (150 mL) and a saturated NaHCO_3_ solution (150 mL). The organic layer was rinsed with a brine solution and dried over anhydrous MgSO_4_. The final residue was purified by vacuum distillation in the presence of 5 (*w/w* %) of hydroquinone to afford BuLA (20.5 g, 75%) as a colorless liquid. ^1^H NMR (400MHz, CDCl_3_, δ): 6.48 (dd, 1H), 6.19 (dd, 1H), 5.90 (dd, 1H), 5.16 (q, 1H), 4.16 (m, 2H), 1.63 (m, 2H), 1.53 (d, 3H), 1.38 (m, 2H), 0.93 (t, 3H); ^13^C NMR (100.6 MHz, CDCl_3_, δ): 170.72, 165.34, 131.74, 127.71, 68.78, 65.13, 30.51, 18.99, 16.96, 13.62. HRMS-TOF for BuLA: [M + NH_4_]^+^ calcd for C_10_H_20_NO_4_^+^, 218.1392 found, 218.1393 [M + H]^+^ calcd for C_10_H_17_O_4_^+^, 201.1127, found, 201.1119; [M + Na]^+^ calcd for C_10_H_16_NaO_4_^+^, 223.0946, found, 223.0942.

### 3.4. General Procedure for the RAFT Polymerization of BuLA Monomer

The synthesis of poly(l-butyl lactate acrylate) (PBuLA) macro-CTA was performed in bulk. A representative procedure is described as follows: AIBN (2.07 mg, 0.01 mmol) 3,5-Bis(2-dodecylthiocarbonothioylthio-1-oxopropoxy)benzoic acid (BTCBA) (or DTPA) (103.3 mg, 0.13 mmol) and l-butyl lactate acrylate (10.0 g, 50.40 mmol) were mixed in a 25 mL Schlenk tube equipped with a teflon stirring bar. The reaction mixture was degassed by bubbling argon for 30 min and subsequently submerged into a preheated, stirring oil bath maintained at 70 °C. To monitor the monomer conversion, the side arm of the tube was purged with argon before it was opened to remove two drops of sample using an airtight syringe. Samples were quenched by immediately placing them into liquid nitrogen exposed to air. After that, samples were dissolved in CDCl_3_ and the monomer conversion was measured by ^1^H NMR spectroscopy. The *M*_n_ and *M*_w_/M_n_ values were determined by GPC calibrated with Poly(methyl methacrylate) (PMMA) standards. Finally, to stop the reaction, the Schlenk flask was placed into nitrogen liquid and opened to air. The final polymerization mixture was dissolved in the minimal volume of DCM and precipitated in 400 mL of cold hexane and isolated by decanting off the supernatant fluid. The procedure was repeated three times and the final polymer was dried under a vacuum for 24 h at room temperature to lead a viscous yellowish liquid. Model homopolymers prepared from butyl, isosorbide acetate and vanillin acrylates were prepared following the same procedure.

### 3.5. General Procedure for RAFT Block Copolymerization Experiments

An illustrative example is provided. CTA-poly(BuLA)-CTA (1.0 g, 0.013 mmol), isosorbide acetate acrylate (0.26 g, 1.06 mmol), AIBN (0.47 mg, 2.86 μmol) (from a dilute stock solution), and 3 mL of ethyl-5-(dimethylamido)-2-methyl-5-oxopentanoate (Rhodiasolv^®^ PolarClean) were added to a Schlenk flask equipped with a teflon stirring bar. The flask was sealed and degassed by bubbling argon for 45 min, and then submerged in a preheated oil bath at 70 °C. After stirring vigorously for 24 h, the flask was immediately submerged into liquid nitrogen and opened to air. Two drops of sample were dissolved in CDCl_3_, and a monomer conversion was determined by ^1^H NMR. The polymerization mixture was dissolved in the minimum of DCM, and precipitated three times in cold methanol (3 × 200 mL). The final copolymer was then dried in a vacuum at room temperature for 24 h. 

### 3.6. Cell Cultures

HT-29 (human colon adenocarcinoma), HeLa (human cervix epithelioid carcinoma) and MIA-Pa-Ca-2 (human Caucasian pancreatic carcinoma) cells were obtained from ATCC and maintained in Dulbecco’s Modified Eagle’s Medium (DMEM) (PAN-Biotech GmbH, Germany) supplemented with 10% FBS (Fetal Bovine Serum), 1% P/S (Penicillin/Streptomycin) and 1% NEAAs (Non-Essential Amino Acids) at 37 °C with 5% CO_2_.

### 3.7. Cell Viability Assays

The cellular cytotoxicity of selected polymers was assessed in three cell lines: HT-29, HeLa and MIA-Pa-Ca-2 cells. The cell viability experiments were carried out by serial dilutions of PBuLA-Z, which was diluted in acetone.

Cells were plated in 96-well plates (8000 cells/95 µL/well in HeLa and MIA-Pa-Ca-2 cells; 9000 cells/95 µL/well in HT-29 cells) with supplemented DMEM without phenol red. Forty-eight hours later, 5 µL of each serial dilutions of the polymer were added to the cells. 100 mg/mL was the maximum tested concentration of the polymer in the cells (poly(butyl lactate acrylate) stock: 2 g/mL). The borders of the plates were in presence of 100 µL of PBS, in order to prevent evaporation-related problems caused by acetone. 

The cells were in the presence of the polymer during 24 h and after this period the cytotoxicity was checked by adding the CellTiter reagent. The CellTiter 96^®^ AQueous One Solution Cell Proliferation Assay Kit (Promega, Madrid, Spain) provides a convenient and sensitive procedure for determining the number of viable cells in cytotoxicity assays. 20 µL of CellTiter reagent (1/4 dilution in DMEM without phenol red) were added into each well of the 96-well assay plates containing the samples. The plates were incubated at 37 °C for 2 h in a humidified, 5% CO_2_ atmosphere and later the absorbance was recorded at 490 nm (background correction at 800 nm). Each experiment was performed in triplicate and repeated at least twice.

### 3.8. General Procedure for 180° Peel Adhesion Tests

The samples for 180° peel adhesion tests were prepared by dissolving the polymer in ethyl acetate to obtain a 30% solid content solution. As a representative example, 200 mg of triblock copolymer was dissolved in 520 μL ethyl acetate. The solution was solvent-cast on a polyethylene terephthalate (PET) film of 19 mm width using a standard laboratory drawdown rod. The film was allowed to dry under ambient conditions open to air in a chemical fume hood for 4 h. The resultant coated film strips were approximately 5 cm long. The peel strength analyses were performed on an Instron 5965 at a peel rate of 300 mm.min^−1^. 5 cm-wide strips of the coated PET films were placed on a clean stainless steel panel, as an adherend. The coated film was gently pressed against steel plate by manually rolling in order to develop good contact between the adhesive and the steel plate. The strip was then peeled from the stainless steel panel. The reported average peel force and standard deviation values were acquired from at least three replicates. Adhesion testing on commercially available scotch 805 PSA was also performed for comparison under identical conditions.

## 4. Conclusions

In summary, our study demonstrates that l-butyl lactate solvent can be upgraded to well-defined tacky triblock copolymers, suitable for applications as PSAs. The functionalization of BuL with an acrylate moiety generated a monomer well behaved under RAFT polymerization. Good control over molecular weight and molecular weight distribution was achieved in bulk, using either monofunctional and bifunctional trithiocarbonate-type chain transfer agents. Subsequently, poly(BuLA), with a relative low *T*_g_ (−20 °C), good thermal stability (5% wt. loss at 340 °C) and low toxicity in the cell cultures assessed was evaluated as sustainable elastomeric core block in all-acrylate ABA copolymers using isosorbide and vanillin-derived glassy polyacrylates as representative end blocks. Materials with a low content of hard building block (<20 wt%) were targeted, pursuing the preparation of materials with high tack. The thermal and mechanical properties of the prepared copolymers were evaluated to demonstrate suitability of rubbery poly(alkyl lactate) building blocks for developing innovative sustainable materials. As is noteworthy, 180° peel adhesion measurements showed that the synthesized biosourced all-acrylic ABA copolymers possess competitive performance when compared with commercial pressure-sensitive tapes. The significance of developing innovative adhesives based on renewable resources combining good thermal stability and low cytotoxicity is that these materials are promising for a broad range of applications, including both biomedical and technological fields. 

## Supplementary Materials

The following are available online: Figure S1: GPC analysis PBuLA-2Z; Figure S2: ^1^H NMR spectrum of the reaction mixture after the chain extension of PBuLA-2Z macro-RAFT agent with IA; Figure S3: GPC traces of the chain extension of PBuLA-2Z macro-RAFT agent with VA; Figure S4: DSC analysis of model homopolymers and block copolymers; Figure S5: DMTA analysis of PVA-PBuLA-PVA triblock; and Figure S6: TGA analysis for PIA-PBuLA-PIA and PVA-PBuLA-PVA under nitrogen and air atmosphere.
