# Supplementary material for: Biosourced All-Acrylic ABA Block Copolymers with Lactic Acid-Based Soft Phase
Source: Molecules. 2020 Dec 5;25(23):5740. doi: 10.3390/molecules25235740 (PMC7731403; doi:10.3390/molecules25235740)
Supplement: Supplementary file 1 [file molecules-25-05740-s001.pdf]

Supplementary Information for

Biosourced All-Acrylic ABA Block Copolymers with Lactic Acid-based Soft Phase

Nabil Bensabeh,[1] Ana Jiménez-Alesanco,[2] Ilme Liblikas,[3] Juan C. Ronda,[1] Virginia Cádiz,[1] Marina Galià,[1] Lauri Vares,[3] Olga Abian,[2,4,5,6,7] Gerard Lligadas[1,*]

[1] Laboratory of Sustainable Polymers, Department of Analytical Chemistry and Organic Chemistry, University Rovira i Virgili, Tarragona 43007, Spain.
[2] Institute for Biocomputation and Physics of Complex Systems (BIFI), Joint Units IQFR-CSIC-BIFI, and GBsC-CSIC-BIFI, Universidad de Zaragoza, Zaragoza 50018, Spain.
[3] Institute of Technology, University of Tartu , Nooruse 1, Tartu 50411, Estonia.
[4] Instituto Aragonés de Ciencias de la Salud (IACS), Zaragoza 50009, Spain.
[5] Instituto de Investigación Sanitaria de Aragón (IIS Aragon), Zaragoza 50009, Spain.
[6] Centro de Investigación Biomédica en Red en el Área Temática de Enfermedades Hepáticas Digestivas (CIBERehd), Madrid 28029, Spain.
[7] Departamento de Bioquímica y Biología Molecular y Celular, Universidad de Zaragoza, Zaragoza 50005, Spain.

*Correspondence: gerard.lligadas@urv.cat; Tel.: +34-977-55-8286

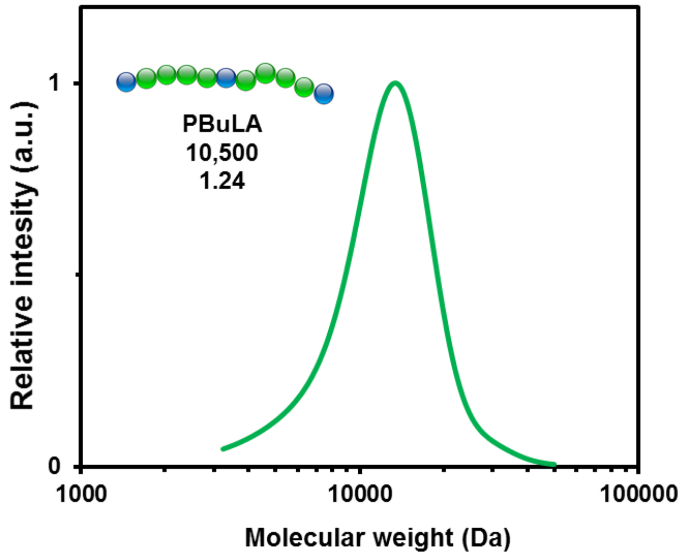

Figure S1. GPC analysis PBuLA-2Z. Numbers shown correspond to $M_{n,GPC}$, and M_w/M_n, from top to bottom.

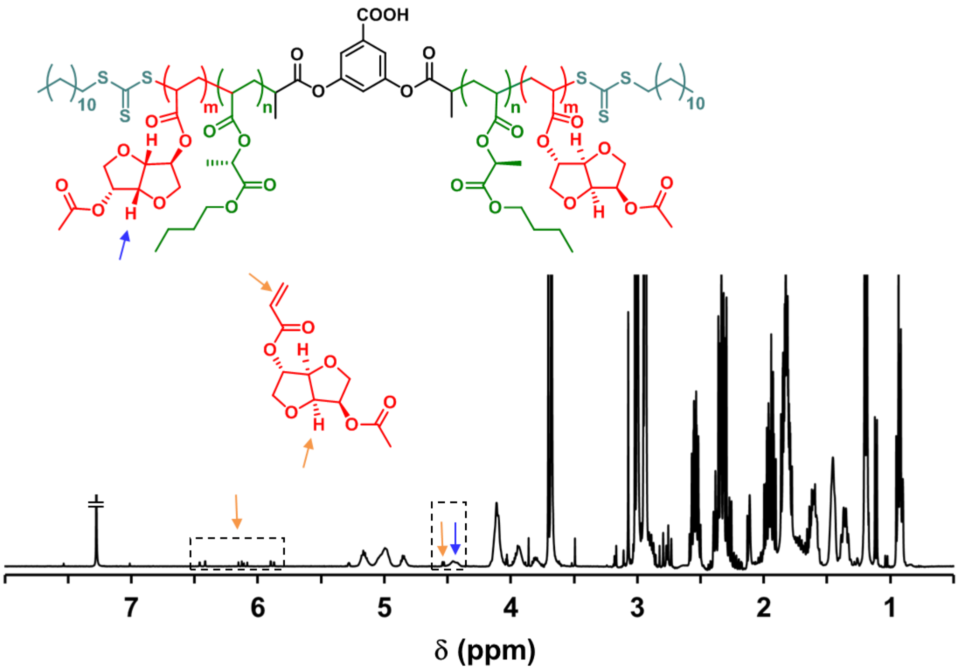

Figure S2. ^{1}H NMR spectrum in $CDCl_3$ of the reaction mixture after the chain extension of PBuLA-2Z macro-RAFT agent with IA monomer at 70 ºC in Rhodiasolv® PolarClean solvent. Monomer conversion was determined to be 86% by integration of the monomer/polymer signals marked with arrows.

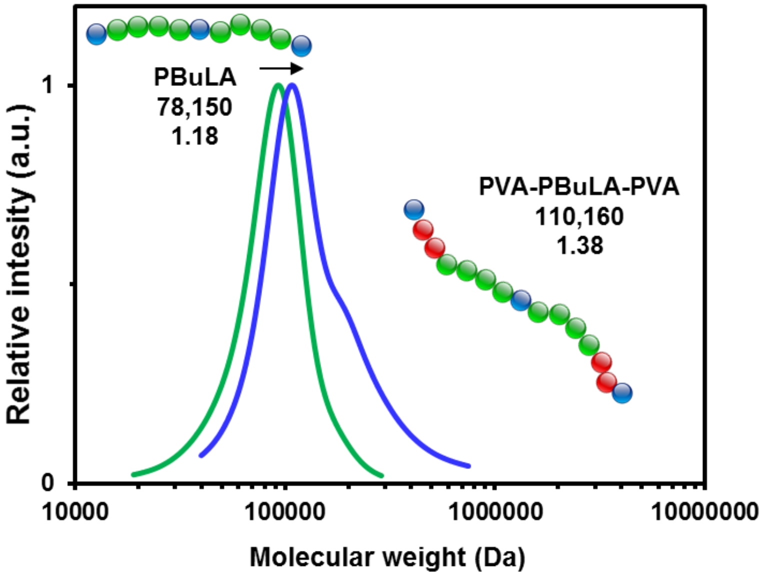

Figure S3. GPC traces of the chain extension of PBuLA-2Z macro-RAFT agent with VA monomer at 70 ºC in Rhodiasolv® PolarClean solvent. Numbers shown correspond to $M_{n,GPC}$, and M_w/M_n, from top to bottom.

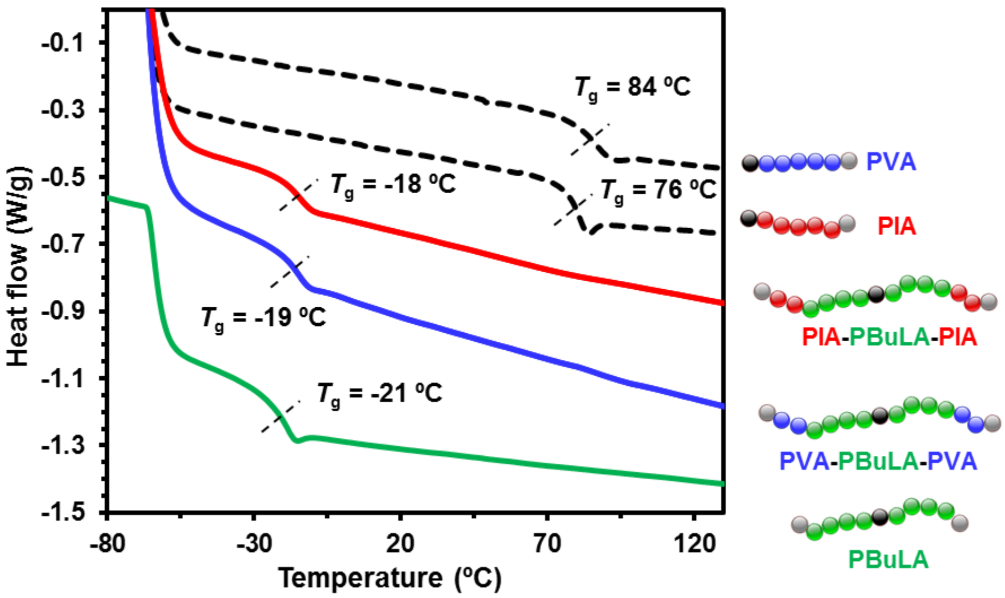

Figure S4. DSC thermograms of PVA ($M_{n,GPC}$ = 14460 g·mol^{-1}), PIA ($M_{n,GPC}$ = 13830 g·mol^{-1}), and PBuLA-2Z ($M_{n,GPC}$ = 78150 g·mol^{-1}) homopolymers and triblock copolymers PIA-PBuLA-PIA ($M_{n,GPC}$ = 103,500 g·mol^{-1}) and PVA-PBuLA-PVA ($M_{n,GPC}$ = 110,150 g·mol^{-1}).

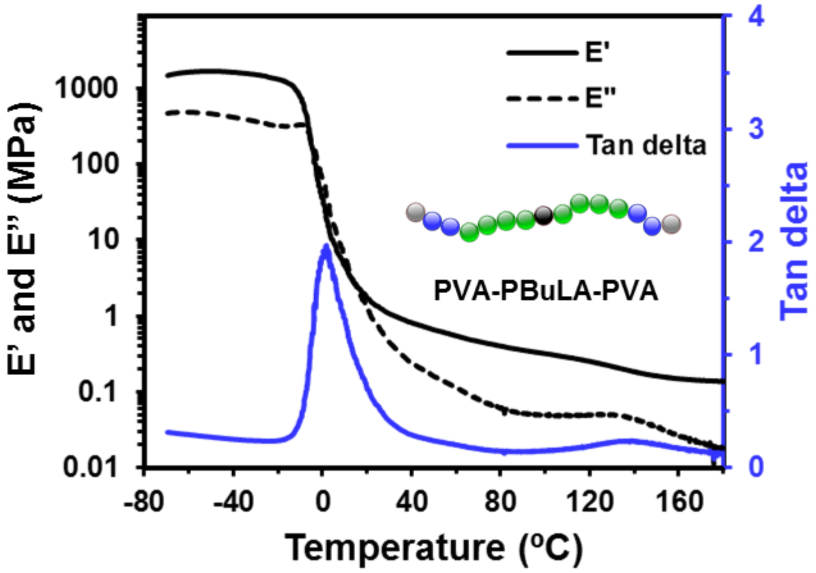

Figure S5. Dynamic tensile storage (E') and loss (E'') moduli and tan δ (= E''/E') as a function of temperature of PVA-PBuLA-PVA triblock.

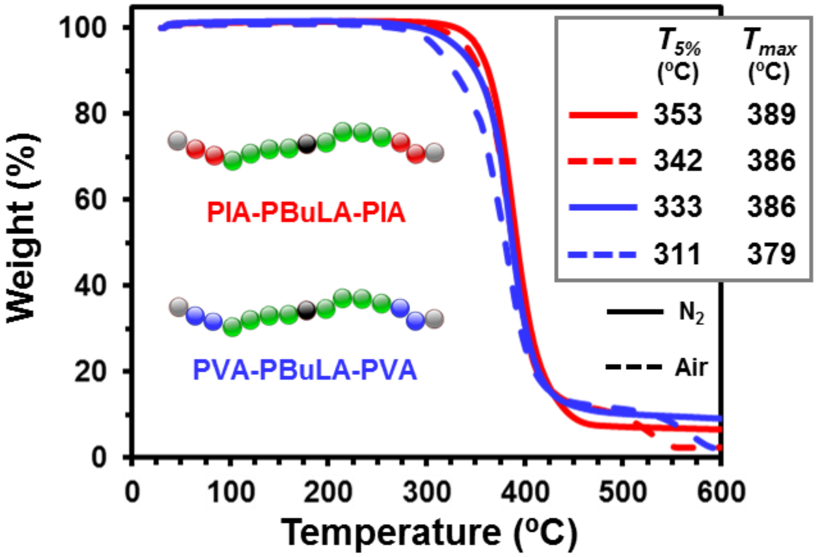

Figure S6. TGA for PIA-PBuLA-PIA (red lines) and PVA-PBuLA-PVA (blue lines) under nitrogen and air atmosphere.
